# *CPSF1* Is Co-Amplified with *MYC* but Is Independently Associated with Alternative Polyadenylation in Cancer

**DOI:** 10.3390/biology14121637

**Published:** 2025-11-21

**Authors:** Abdulrahman A. Alahmari

**Affiliations:** Department of Medical Laboratory, College of Applied Medical Sciences, Prince Sattam Bin Abdulaziz University, Alkharj 11942, Saudi Arabia; aa.alahmari@psau.edu.sa

**Keywords:** cleavage and polyadenylation, mutations, copy number variations, *CPSF1*, *MYC*, cancer prognosis, 3′UTR, alternative polyadenylation

## Abstract

Cleavage and polyadenylation (CPA) is a co-transcriptional mRNA processing mechanism that is central to mRNA and protein function. Dysregulation of CPA is widespread in cancer, promotes oncogenic programs, and affects patient outcomes. The CPA machinery is composed of multiple factors, and while prior research has investigated the impact of CPA gene expression on cancer phenotypes, the contribution of genomic alterations, such as mutations and copy number variations, remains largely unexplored. In this study, we conducted a pan-cancer analysis of genomic alterations in CPA genes. While numerous CPA genes harbor somatic mutations, these mutations do not significantly impact mRNA expression or provide prognostic value. In contrast, we found that copy number alterations in CPA genes have substantial clinical relevance. Notably, we identified the cleavage and polyadenylation specificity factor 1 (*CPSF1*) gene as the most frequently amplified CPA gene in cancer. While amplification of *CPSF1* and *MYC* are co-occuring, *CPSF1* amplification independently correlates with poor prognosis. We also found that *CPSF1* amplification can impact 3′UTR length regardless of *MYC* status. Our study highlights the importance of *CPSF1* as a promising prognostic factor in cancer and as a therapeutic intervention target to study in the future.

## Simple Summary

This study investigates the genomic alterations of cleavage and polyadenylation factors and their clinical relevance in cancer. We show that mutations in CPA genes may not be clinically relevant. However, copy number variations, particularly amplification of *CPSF1*, demonstrate significant impact on patient prognosis. We show that *CPSF1* amplification, although co-occurring with *MYC* amplification, can independently change alternative polyadenylation (APA) patterns and dysregulate cancer-associated gene signatures. These results highlight the crucial impact of CPA gene copy number variation on biological processes and clinical outcomes.

## 1. Introduction

Cleavage and polyadenylation (CPA) is a co-transcriptional mRNA process that is crucial for mRNA maturity and expression [1]. CPA begins with an endonucleolytic cleavage of the pre-mRNA followed by an addition of a poly(A) tail at the 3′ end of the transcript. This process is largely regulated by a multi-protein complex that recognizes a polyadenylation signal (PAS) and catalyzes the endonucleolytic cleavage of pre-mRNA transcripts. The CPA complex is composed of the cleavage and polyadenylation specificity factor (CPSF) complex, the cleavage stimulation factor (CSTF) complex, the cleavage factor (CFIm and CFIIm) complexes, and other auxiliary factors [2]. The majority of pre-mRNAs have more than one PAS, which determines where pre-mRNAs are cleaved, thus leading to the production of mRNAs with the same coding region but with different 3′ end lengths, a process known as alternative polyadenylation [3].

Alternative polyadenylation, or APA, has recently gained recognition as an important modulator of gene dysregulation in cancer [4]. APA is widely dysregulated in cancer, mainly by a global shortening of the 3′ ends of many oncogenes leading to their overexpression across multiple tumor types [5,6,7]. This global shift in 3′ end lengths is accompanied by mRNA expression dysregulation of many CPA factors. For example, *CPSF1* and *CPSF3* are upregulated in liver and pancreatic cancer, respectively, and are associated with poor patient survival [8,9]. Conversely, *NUDT21* is significantly downregulated in glioblastoma and is associated with increased tumorigenicity and unfavorable outcomes [10,11,12]. While many studies have shown the profound implications of CPA gene dysregulation at the expression level in tumorigenesis, it is still unknown whether CPA genes are altered at a genomic level and whether such alterations have biological and clinical implications. Several studies have investigated mutations in the polyadenylation signal sequences globally, but not mutations of CPA genes. For instance, single nucleotide polymorphism (SNP) in the PAS sequence, although rare, can impact cleavage efficiency [13,14]. Importantly, mutations that affect 3′-end PAS were found to be enriched in tumor suppressor genes in cancer [14]. These mutations have a profound impact on the expression of tumor suppressor genes, mainly leading to their downregulation [14]. These studies indicate that genomic alterations can affect cleavage and polyadenylation function. Nevertheless, genomic alterations of the CPA machinery itself have not been investigated.

Here, we conduct a pan-cancer analysis of genomic alterations in the CPA genes. We show that mutations of CPA genes are not very common in cancer and do not impact patient outcome. However, genomic alterations at the copy number level affect many CPA genes. Of note, amplification of CPA genes is more frequent and is clinically relevant. We specifically identify cleavage and polyadenylation specificity factor 1 (*CPSF1*) as the most frequently amplified CPA gene across cancers. Importantly, *CPSF1* amplification co-occurs with *MYC* amplification but is an independent predictor of poor prognosis. Finally, we find that *CPSF1* amplification is associated with alternative polyadenylation in cancer. These findings highlight the pivotal impact of CPA gene copy number alterations in cancer.

## 2. Materials and Methods

### 2.1. Analysis Cohorts

The TCGA Pan-Cancer Atlas public somatic mutation data (mc3.v0.2.8.PUBLIC.maf.gz) was obtained from the Genomic Data Commons (GDC) (https://gdc.cancer.gov/about-data/publications/pancanatlas) (accessed on 12 December 2024). cBioPortal for cancer genomics (www.cbioportal.org) (accessed on 25 January 2025) [15] was used to download the following data: (1) the mRNA expression data (RSEM; Batch normalized from Illumina HiSeq_RNASeqV2, log2[value + 1]), (2) the Cancer Cell Line Encyclopedia [16,17] mutation and protein expression data sets, and (3) the gene-level copy number variation (CNV) data (GISTIC2) [18]. Clinical data were downloaded from the UCSC Xena Browser (https://xenabrowser.net) (accessed on 25 January 2025) [19]. Alternative polyadenylation (APA) usage data for 32 tumor types were obtained from The Cancer 3′UTR Atlas (TC3A) repository (https://github.com/CHENCANcc/TC3A_PDUI) (accessed on 5 August 2025) [20]. Z-scores of mRNA expression used to integrate with APA results were downloaded from the TCGA cBioPortal datahub (https://github.com/cBioPortal/datahub) (accessed on 5 August 2025).

### 2.2. Somatic Mutation Analysis

The Mutation Annotation Format (MAF) file derived from the TCGA MC3 project provides harmonized and quality-filtered somatic mutation data across multiple cancer types. We analyzed the MAF file using the maftools R package [21] to generate mutation oncoplots showing the somatic mutation rate and lollipop plots showing the distribution of mutation variants across different gene domains.

### 2.3. Copy Number Variation Analysis

GISTIC2 gene-level CNV data were obtained from cBioPortal and processed to generate a CNV alteration matrix. The input file was filtered to remove non-numeric entries to ensure compatibility with matrix transformation. The CNV matrix was transposed and converted to a gene-by-sample matrix where copy number variants were encoded as indicated here: “2” = Amplification (high amplification levels), “1” = Gain (low amplification levels), “0” = Diploid (normal copy number), “−1” = Shallow (heterozygous) deletion, and “−2” = Deep (homozygous) deletion. The frequency of all alterations was computed by counting non-zero entries across all samples for each gene. The frequency of specific alterations was computed by counting only the corresponding entries across all samples for each gene. For mutual exclusivity, co-occurrence q-values were obtained directly from cBioPortal.

### 2.4. Correlation Between Expression and Mutation

For mRNA expression data, raw mRNA expression values were downloaded from cBioPortal and transformed using a log_2_ scale (log_2_(expression + 1)). Cancer Cell Line Encyclopedia (CCLE) Z-score normalized protein expression levels were downloaded from the cBioPortal. Both mRNA and protein expression data were categorized into high and low expression based on median expression values. The association between mutation status and expression was then assessed and visualized using a boxplot stratified by mutation status. Data preprocessing and visualization were performed in R (version 4.2.0) using the tidyverse (v2.0.0) and ggplot2 (v3.5.0) packages.

### 2.5. Alternative Polyadenylation (APA) Analysis

Gene-level APA was quantified using the percentage of distal poly(A) site usage index (PDUI) derived from TC3A (The Cancer 3′UTR Atlas) [12]. TC3A provides PDUI values computed from TCGA RNA-seq data using the DaPars algorithm as previously described [6]. PDUI represents the proportion of distal 3′ poly(A) site usage for each gene per sample. Higher values of PDUI indicate preferential usage of distal poly(A) sites and thus 3′UTR lengthening, whereas lower PDUI values correspond to shorter 3′UTRs. To identify APA events associated with copy number status of *CPSF1*, we compared Percent Distal Usage Index (PDUI) values between high (≥75th percentile) and low (≤25th percentile) *CPSF1* copy number groups using a Wilcoxon rank-sum test. Differences in median PDUI between groups (ΔPDUI) were computed for each gene as follows: ΔPDUI = median(PDUI^CPSF1-high^) − median(PDUI^CPSF1-low^). Adjusted *p*-values were obtained using the Benjamini–Hochberg (BH) method. Genes with adjusted *p* < 0.05 were considered significantly APA altered. To integrate APA and mRNA expression data, mean Z-scores were calculated for each group per gene and differences in Z-score means (ΔZ-score) between high and low *CPSF1* copy number groups were computed per gene. Samples where amplification of *CPSF1* and *MYC* are co-occurring were excluded, and only *MYC* diploid samples were included.

### 2.6. Pathway Enrichment Analysis

Gene sets for each quadrant were subjected to pathway enrichment analysis using the enrichR R package (version 3.4) [22,23,24]. The MSigDB Hallmark gene set was used as a reference database [25]. EnrichR-implemented Fisher’s exact test was used to evaluate enrichment significance. The Benjamini–Hochberg (BH) method was used to adjust *p*-values for multiple comparisons. Enriched pathways with adjusted *p* < 0.05 were considered statistically significant.

### 2.7. Survival Analysis

Overall survival (OS) and progression-free survival (PFS) data were downloaded from the UCSC Xena Browser [19]. Kaplan–Meier curves were generated using the “survival” and “survminer” R packages to determine the association between genomic alterations and clinical outcomes. A log-rank test was performed to compare survival outcomes between groups, and a *p*-value < 0.05 was considered statistically significant. All analyses were performed in R (version 4.2.0).

## 3. Results

### 3.1. CPA Mutational Landscape Across TCGA Cancer Types

**Figure 1:**
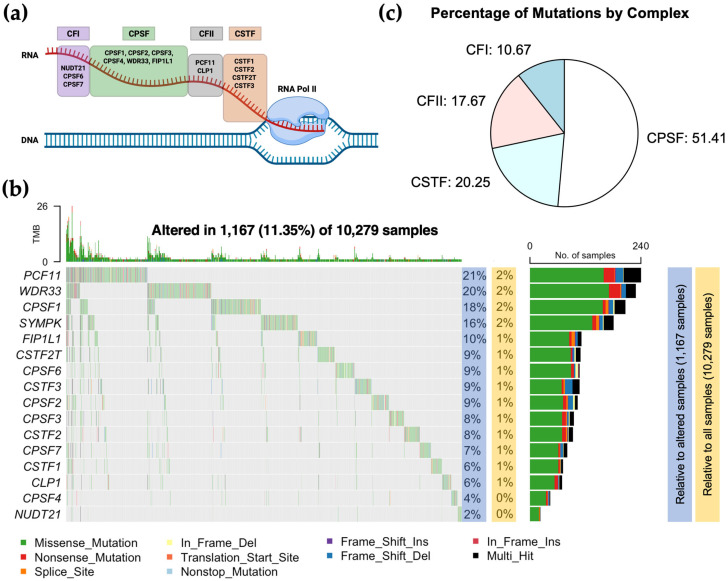
Mutational landscape of cleavage and polyadenylation genes in pan-cancer. (**a**), Schematic of CPA complex subunits binding to the nascent RNA transcript. Orange: cleavage stimulation factor (CSTF) subcomplex, Gray: mammalian cleavage factor I (CFIm) subcomplex, Green: cleavage and polyadenylation specificity factor (CPSF) subcomplex, Purple: mammalian cleavage factor II (CFIIm) subcomplex. (**b**), Oncoplot showing the mutational landscape of 16 CPA genes across 33 cancer types from TCGA. Percentages highlighted in blue represent mutation rate relative to samples with alterations in any CPA gene, while those highlighted in yellow indicate mutation rate relative to all samples with or without CPA gene mutation. Types of mutations are color-coded below the oncoplot. (**c**), Pie chart showing the mutation percentage by CPA subcomplex.

The CPA machinery is composed of multiple complexes including the cleavage and polyadenylation specificity factor (CPSF) complex, the cleavage stimulation factor (CSTF) complex, the cleavage factor (CFIm and CFIIm) complexes, and other auxiliary factors (Figure 1a). The CPSF complex is composed of CPSF1, CPSF2, CPSF3, CPSF4, WDR33, SYMPK, and FIP1L1. This complex recognizes the polyadenylation signal (PAS) and contains the enzyme that catalyzes mRNA cleavage (CPSF3). The CSTF complex is composed of CSTF1, CSTF2, CSTF2T, and CSTF3. This complex binds a U/GU-rich sequence element downstream of the polyadenylation cleavage site and stabilizes the CPA complex. The CFIm complex is composed of NUDT21, CPSF6, and CPSF7 and can directly bind to the UGUA motifs near the PAS. The CFIIm binds to G-rich sequence elements and consists of two CPA factors, CLP1 and PCF11. The CFIm and CFIIm complexes promote distal and proximal PAS usage, respectively, and therefore are crucial for alternative polyadenylation. To analyze CPA gene mutations across cancer types, we interrogated publicly available cancer genomic data from The Cancer Genome Atlas (TCGA) (n = 10,279 subjects) [26]. We utilized the maftools R package to find somatic mutations in the CPA core genes [21]. From the 10,279 analyzed subjects, 11.35% (1167 subjects, referred to from now on as altered subjects) had mutations in many CPA genes (Figure 1b). *PCF11*, *WDR33*, *CPSF1*, and *SYMPK* were the top four mutated genes accounting for 20.56%, 19.62%, 17.73%, and 15.5%, respectively, from altered subjects (Figure 1b, blue-highlighted). The other CPA genes were each mutated in less than 10% of altered subjects (Figure 1b, blue-highlighted). When accounting for all subjects (with both wild type and mutated CPA genes), *PCF11* was mutated in 2.33% of patients followed by *WDR33* (2.22%) and *CPSF1* (2.01%) (Figure 1b, orange-highlighted). Among the four CPA complexes, the CPSF complex had the highest mutation rate (51.41%) (Figure 1c). The CSTF, CFI, and CFII complexes had mutation rates of 20.25%, 17.67%, and 10.67, respectively (Figure 1c).

### 3.2. CPA Mutations Are Not Associated with Gene Expression Changes

**Figure 2:**
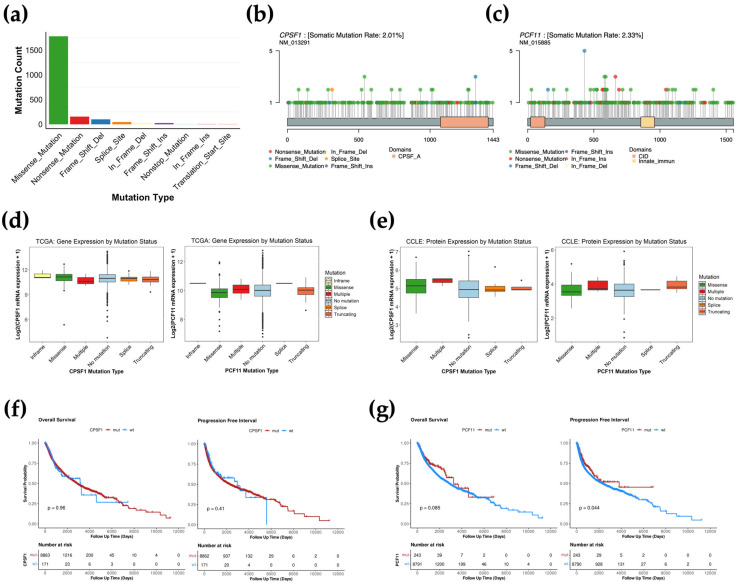
Impact of CPA mutation on gene expression and prognosis. (**a**), CPA mutation burden across various variant classes. (**b**,**c**), Lollipop plot showing the distribution of mutations across the *CPSF1* (**b**) and *PCF11* (**c**) gene domains. The Y-axis indicates the number of mutations for each specific variant. (**d**), The association between different variant classes and *CPSF1* and *PCF11* TCGA gene expression. (**e**), The association between different variant classes and CPSF1 and PCF11 CCLE protein expression. (**f**,**g**), Kaplan–Meier curves showing overall and progression-free survival between wild type and mutant *CPSF1* (**f**) and *PCF11* (**g**) in pan-cancer. “Blue” indicates patients with wild type genes while “Red” indicates patients with mutations.

Somatic mutations contribute to cancer phenotypes by disrupting gene or protein expression, thereby affecting cellular behavior. Given that different types of mutations can result in distinct molecular changes, we first quantified the mutation burden across various variant classes. Missense mutations were the most common mutation type with a total of 1456 occurrences, accounting for 61.6% of total mutation counts (Figure 2a, Supplementary Table S1). The majority of these mutations were found in the CPSF and CSTF CPA genes (Supplementary Table S1). The second most common mutation type was nonsense mutations with 624 occurrences, accounting for 26.4% of total mutation counts (Figure 2a, Supplementary Table S1). Most of these nonsense mutation occurrences were found in the CFI and CFII category (Supplementary Table S1). The other mutation types combined were less frequent (less than 12% of total mutation counts) across all CPA groups (Figure 2a, Supplementary Table S1). We then sought to determine the frequency of different variants across the domains of the most mutated CPA genes. Intriguingly, mutations were not clustered in any domain or region of any of the genes analyzed (Figure 2b,c, Supplementary Figure S1). This suggests that mutations of the CPA genes randomly occur, possibly because of increased tumor mutation burden, and are not necessarily driver mutations. Therefore, we sought to determine whether mutations in CPA genes affect RNA or protein functions. We analyzed the relationship between different types of mutations and their effects on the gene expression of the *CPSF1* and *PCF11* CPA genes. Interestingly, no mutation types were associated with changes in mRNA expression (Figure 2d). In addition, we analyzed the relationship between CPA gene mutations and their effects on protein expression using the Cancer Cell Line Encyclopedia (CCLE) database. Similar to mRNA expression, there was no association between protein expression and *CPSF1* or *PCF11* mutations in cancer cell lines (Figure 2e). To further investigate whether these mutations are clinically relevant, we assessed their correlation with patient outcome. We found that *CPSF1* and *PCF11* mutations do not predict progression-free or overall survival of cancer patients (Figure 2f,g). Mutations in other CPA genes, specifically *WDR33*, are also not associated with significant changes in patient prognosis (Supplementary Figure S2). Overall, these data suggest that CPA mutations do not significantly impact cancer outcomes.

### 3.3. CPSF1 Is the Most Amplified CPA Gene in Cancer

**Figure 3:**
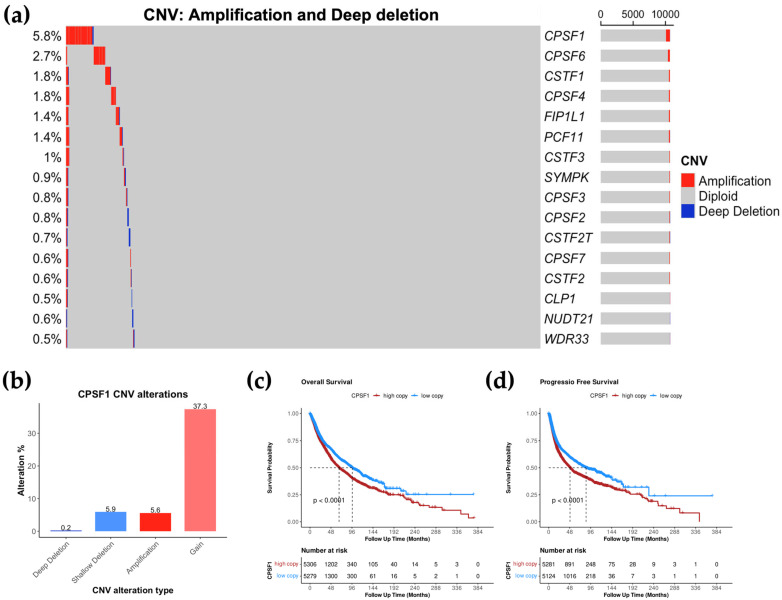
Copy number alterations in CPA genes and their clinical relevance. (**a**) Oncoplot showing the percentages of copy number variations (CNVs) in 16 CPA genes across 33 cancer types from TCGA. CNV here includes amplification (red) and deep deletion (blue). Diploid (gray) denotes normal copy number. (**b**), Alteration rate of *CPSF1* copy number variants in pan-cancer. (**c**,**d**), Kaplan–Meier curves showing overall (**c**) and progression-free (**d**) survival between high and low *CPSF1* copy number in pan-cancer.

We next sought to assess whether CPA genes exhibit other genomic changes, specifically copy number variation. We used the TCGA pan-cancer copy number data from cBioPortal [15,26]. These data contain copy number status data including amplification, gain, deep deletion, shallow deletion, and diploidy. “Amplification” indicates high amplification levels, while “gain” represents low amplification levels. “Deep deletion” refers to homozygous deletion (loss of two alleles), while “shallow deletion” refers to heterozygous deletion (loss of one allele). Among the CPA genes analyzed in this study, *CPSF1*, *CSTF1*, and *CPSF4* were the top three CPA genes with copy number alterations accounting for 49%, 42%, and 42.8%, respectively (Supplementary Figure S3A). The other CPA genes were each altered in less than 31% of patients. We then analyzed CPA gene copy number at the levels of amplification and deep deletion as these copy number alterations are associated with oncogenic activities in cancer. *CPSF1* was the CPA gene with the highest copy number alterations at the levels of amplification and deep deletion (5.8%) (Figure 3a). Importantly, amplification of *CPSF1* accounted for the majority of these copy number changes with 5.6%, while deep deletion occured in only 0.2% of patients (Table 1). This is also the case at the levels of copy number gain or shallow deletion, which occur in 43.2% of patients (Supplementary Figure S3B). However, the majority of these changes are attributed to *CPSF1* copy number gain, with shallow deletion of *CPSF1* accounting for only 5.9% of patients (Table 1). *CPSF6*, *CSTF1* and *CPSF4* were also among the top most amplified CPA genes (2.7%, 1.8%, and 1.8%, respectively) with no deep deletion (Figure 3a, Table 1). Therefore, *CPSF1* is the most commonly amplified CPA gene in cancer.

### 3.4. High CPSF1 Copy Number Is Associated with Poor Prognosis

To determine whether *CPSF1* copy number variations are clinically relevant, we analyzed the TCGA gene-level copy number (GISTIC2) data in relation to patient survival outcomes. Even though *CPSF1* is amplified (high amplification levels) in only 5.6% of patients, a large proportion of patients (37.3%) still have *CPSF1* gain (low amplification levels) (Figure 3b). Therefore, we classified patients into two groups based on GISTIC2 copy number values: high *CPSF1* copy number (above GISTIC2 median) and low *CPSF1* copy number (below GISTIC2 median). Statistical significance was then assessed using the log-rank test, and Kaplan–Meier survival curves were generated to compare survival outcomes between these groups. We found that high *CPSF1* copy number is associated with poor outcomes. Specifically, patients with high *CPSF1* copy number had a median overall survival of 66.4 months, while those with low *CPSF1* copy number had a median overall survival of 96.6 months (Figure 3c,d). Also, patients with high *CPSF1* copy number had a median progression-free survival of 48.6 months, while those with low *CPSF1* copy number had a median progression-free survival of 85.4 months (Figure 3c,d). These results suggest that high *CPSF1* copy number may serve as a prognostic indicator for patient outcomes.

### 3.5. Amplification of CPSF1 Co-Occurs with MYC Amplification but Is an Independent Prognostic Factor

**Figure 4:**
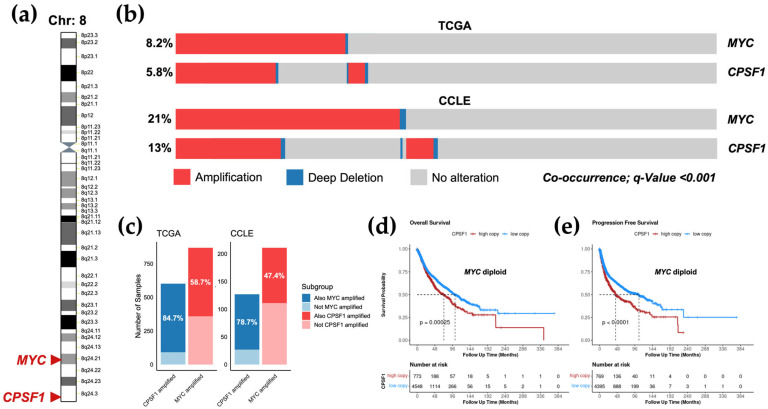
Co-occurrence of *CPSF1* and *MYC* amplification in cancer. (**a**), Genomic location of *CPSF1* and *MYC* at chromosome 8. (**b**), The co-occurrence of *CPSF1* and *MYC* copy number alterations in TCGA (top panel) and CCLE (bottom panel). Only amplification (red) and deep deletion (blue) were included in this analysis. Gray indicates diploid or low-level alterations (gain and shallow deletion). (**c**), Stacked bar plots showing the percentage of *CPSF1* and *MYC* amplification overlaps in TCGA (left panel) and CCLE (right panel). (**d**,**e**), Kaplan–Meier curves showing overall (**d**) and progression-free (**e**) survival between high (above median) and low (below median) *CPSF1* copy number in patients with diploid *MYC*.

We next asked whether amplification of *CPSF1* is independently occurring or is affected by other amplified genomic regions. *CPSF1* is located on chromosome 8 at the 8q24 region, which is the same region where *MYC* is located (Figure 4a). This region is the most amplified region in multiple cancer types [27,28]. Importantly, *MYC* is highly oncogenic and is associated with unfavorable prognosis in multiple cancer types [28,29,30]. We found that *CPSF1* amplification co-occurs with *MYC* amplification in both patients and cancer cell lines (Figure 4b). Specifically, 84.7% of TCGA subjects with *CPSF1* amplification also had *MYC* amplification, while 58.7% of *MYC*-amplified subjects had *CPSF1* amplification (Figure 4c). In CCLE, *MYC* was amplified in 78.7% of *CPSF1*-amplified cells, while 47.4% of *MYC*-amplified cells had *CPSF1* amplification (Figure 4c). We next sought to determine whether *CPSF1* predicts patient prognosis independent of *MYC*. We therefore focused our analysis on patients with no *MYC* copy number alterations. *MYC*-nondiploid patients were first filtered out, leaving only patients with diploid (normal) *MYC* copy number. We then stratified patients into two groups based on GISTIC2 copy number values, high *CPSF1* copy number (above GISTIC2 median) and low *CPSF1* copy number (below GISTIC2 median), and generated Kaplan–Meier survival curves to compare survival outcomes. We found that high *CPSF1* copy number is associated with poor outcomes. Specifically, patients with high *CPSF1* expression had a median overall survival of 72.7 months, while those with low *CPSF1* expression had a median overall survival of 103.2 months (Figure 4d). Also, patients with high *CPSF1* expression had a median progression-free survival of 43.9 months, while those with low *CPSF1* expression had a median progression-free survival of 107.4 months (Figure 4e). These results suggest that high *CPSF1* copy number serves as a prognostic marker in cancer independent of *MYC*.

### 3.6. CPSF1 Amplification Is Associated with Alternative Polyadenylation Events in Cancer

**Figure 5:**
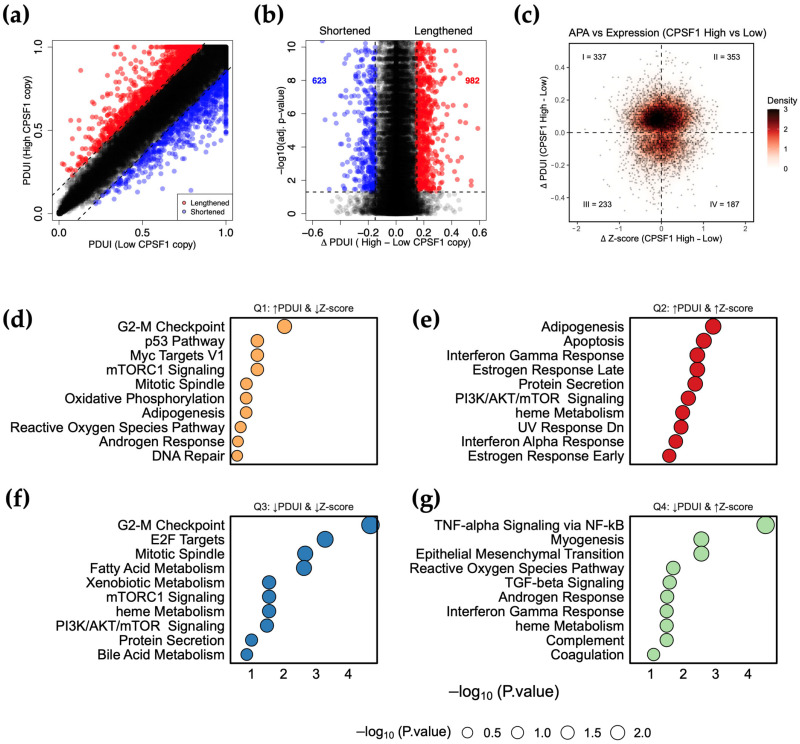
Alternative polyadenylation patterns in *CPSF1* amplified cancers. (**a**), A dot plot showing PDUI score of each gene in high and low *CPSF1* copy number. Dashed lines represent 0.15 cutoffs. Blue dots represent 3′-UTR-shortened genes while red dots represent 3′-UTR-lengthened genes. (**b**), A volcano plot denoting 3′-UTR-lengthened (red) and 3′-UTR-shortened (blue) genes (ΔPDUI = ±0.15; Adjusted *p*-value < 0.05). (**c**), Quadrant dot plot of ΔPDUI (*y*-axis) and ΔZ-scores (*x*-axis). (**d**–**g**), Pathway enrichment analysis of different quadrants from panel C. Upward and downward arrows represent an increase and a decrease, respectively.

To determine whether *CPSF1* amplification is associated with APA events, we utilized the APA usage data from The Cancer 3′UTR Atlas (TC3A) containing Percent Distal Usage Index (PDUI) values for each gene. To identify APA events, we quantified the change in PDUI (ΔPDUI) between high and low *CPSF1* copy number groups in *MYC*-diploid samples. A positive index of ΔPDUI indicates 3′UTR lengthening, while a negative index indicates 3′UTR shortening. We chose ΔPDUI = ±0.15 as a threshold to minimize false positive/negative APA events. To determine the extent of 3′-UTR shortening and lengthening, we compared the PDUI scores for each gene between high and low *CPSF1* copy number samples (Figure 5a,b). While many genes do not undergo changes in APA (gray/black dots), *CPSF1* amplification is associated with significant 3′-UTR shortening events (blue dots, n = 623) and significant lengthening events (red dots, n = 982) (Figure 5b, Supplementary Figure S4). To determine if these APA events were associated with changes in gene expression, we calculated the differences in Z-score means (ΔZ-score) per gene between high and low *CPSF1* copy number groups. Many APA-altered genes were indeed associated with gene expression changes and are represented in four quadrants based on ΔPDUI and ΔZ-score (Figure 5c). The first quadrant (Q1) represents genes with APA lengthening events (ΔPDUI ≥ 0.15) and low gene expression (ΔZ-score < 0). The second quadrant (Q2) represents genes with APA lengthening events (ΔPDUI ≥ 0.15) and high gene expression (ΔZ-score > 0). The third quadrant (Q3) represents genes with APA shortening events (ΔPDUI ≤ 0.15) and low gene expression (ΔZ-score < 0). The fourth quadrant (Q4) represents genes with APA shortening events (ΔPDUI ≤ 0.15) and high gene expression (ΔZ-score > 0) (Figure 5c). To determine whether genes in each quadrant fall under a specific pathway that might be associated with cancer, we used the MSigDB (Human Molecular Signatures Database) Hallmark gene set within the enrichR R package to enrich for pathway terms. Distinct biological signatures were associated with each quadrant (Figure 5d–g). For example, Q1 genes were enriched for the p53 pathway suggesting reduced tumor-suppressive activity upon 3′UTR lengthening (Figure 5d). In contrast, Q4 genes were enriched for pathways such as epithelial to mesenchymal transition suggesting increased metastatic behavior upon 3′UTR shortening (Figure 5g). Other pathways, such as G2-M Checkpoint (Figure 5d,f), were enriched in multiple quadrants, suggesting that *CPSF1* amplification could impact oncogenic activity by regulating gene expression or APA independently. In general, 3′UTR lengthening and shortening are associated with decreased and increased gene expression, respectively. However, APA shift could impact gene expression in either direction. These data suggest that *CPSF1* amplification can influence APA patterns and gene expression, collectively or individually, thus affecting cancer behavior.

## 4. Discussion

Our study advances beyond previous pan-cancer analyses of mRNA processing and alternative polyadenylation in several ways. First, while prior studies have centered their analyses on the interaction between CPA and transcriptional dysregulation, we here interrogate the genomic alterations that occur in the CPA machinery itself and their link to APA dysregulation and clinical outcomes. Second, the impact of CPA gene co-localization with frequently amplified genes has never been investigated. Third, the link between CPA copy number variation, APA, and gene expression has not been established before. To our knowledge, our study is the first to perform a large analysis of CPA gene alterations in cancer. We performed a comprehensive analysis of genomic alterations in cleavage and polyadenylation genes in 33 cancer types. While somatic mutations of CPA genes lack clinical relevance, copy number amplifications emerge as a potential prognostic factor in cancer.

Somatic mutations allow transformation of normal cells into cancer cells and therefore are a leading cause of cancer [31]. However, many genes may have mutations yet are not affected functionally [32]. Here, we investigated the frequency of CPA genes’ mutations and whether they are biologically or clinically significant. We found that many CPA genes are mutated in cancer, notably *PCF11*, *WDR33*, *CPSF1*, and *SYMPK*. These mutations are predominantly missense mutations, raising the possibility of CPA gene and protein dysregulation. However, there was no correlation between CPA mutations and the expression at a gene or protein level. This result is not surprising, as the correlation between mutations and gene or protein expression is not universally applicable. In fact, many genes may possess mutations that affect mRNA or protein function without dysregulating their expression levels [33]. Importantly, we found that CPA mutations are distributed across multiple CPA gene domains and have low frequency. In addition, these mutations do not predict survival probability in cancer patients. These results drive the conclusion that mutations in the CPA genes may have little to no influence on cancer. However, this does not rule out the possibility that specific variants may have a biological or clinical significance. For example, a pathogenic variant of the CPA gene *CLP1* was found to alter mRNA processing patterns in neurodegeneration disease models [34]. Another group has reported multiple novel *CPSF1* mutations that are associated with early-onset high myopia, although causality has not been investigated [35]. Furthermore, homozygous missense variants in *CPSF3* show severe symptoms of intellectual disability syndrome, while heterozygous carriers of the same variant lack these symptoms [36]. These studies highlight the potential of specific CPA variants in disease etiology. Therefore, the pathogenicity of specific CPA variants warrants further investigation.

Unlike mutations, copy number variations (i.e., amplifications and deletions) influence tumorigenicity by affecting large regions of the genome rather than single nucleotides [37,38]. However, the status of CPA gene copy number and whether it is associated with patient outcome has never been investigated. We found that many CPA genes are recurrently altered at the copy number level. Specifically, *CPSF1* was the most amplified CPA gene in cancer. Aberrant expression of *CPSF1* has been found to alter polyadenylation patterns, promote cell proliferation, and increase survival in many cancer cell line models [8,39,40,41,42,43]. *CPSF1* is highly upregulated in these cancer models, and knockdown of endogenous *CPSF1* levels decreased cancer cell proliferation and survival. These data strongly indicate that *CPSF1* has a potential role in cancer. Copy number of other CPA genes is altered less frequently. *CPSF6*, for instance, is the second most amplified CPA gene in cancer. Importantly, *CPSF6* has been found to promote cancer progression, while its depletion decreases cancer progression, dysregulates mRNA processing, and enhances antiviral immune responses [44,45,46,47]. These data indicate that CPA genes possess an oncogenic role in cancer. Importantly, gene amplification increases copy number, and therefore mRNA expression may drastically increase. CPSF1 is responsible for recognition of the polyadenylation signal (PAS), thus initiating the process of 3′ end processing. PAS recognition allows other CPA factors to assemble and bind RNA. Increased abundance of CPSF1 can influence CPA complex stability, formation, and recognition of PASs. This may affect which PAS is recognized and bound by CPA factors as affinity to bind canonical PAS changes eventually resulting in APA. However, canonical PASs can be anywhere in the 3′UTR and not necessarily at proximal sites. Increased dosage of CPSF1 may increase CPA complex engagement with proximal PASs even if they are non-canonical PASs. This leads to 3′UTR shortening and therefore loss of miRNA/RBP regulatory elements, which can promote oncogenic programs in cancer. While many studies have elucidated CPA gene expression potential in cancer progression, further studies are needed to experimentally investigate the role of CPA copy number alterations in cancer models.

Given the fact that *CPSF1* is the most amplified CPA gene in cancer, it is crucial to study its clinical and biological significance. While *CPSF1* amplification occurs in only 5.6% of cancer patients, *CPSF1* remains on the top list of CPA genes with copy number gain in around 37.3% of patients. We found that high *CPSF1* copy number is significantly associated with poor overall and progression-free survival outcomes. This is consistent with previous reports where high *CPSF1* expression predicted unfavorable outcomes in multiple cancer types [8,40,43]. Interestingly, *CPSF1* is located at chromosome region 8q24, the same genomic region known for harboring the *MYC* oncogene [27,48]. *MYC* is the most amplified gene in cancer and is associated with poor outcomes [28,29,30]. We show that *CPSF1* amplification frequently, but not always, co-occurs with *MYC* amplification in patients and cancer cell lines. These data raise the possibility that clinical association with *CPSF1* amplification may be due to *MYC* oncogenic activity. However, when *MYC* amplification is excluded from our analysis, we find that *CPSF1* amplification alone can predict patient prognosis. This is consistent with a previous study where amplification of the 8q21 region was associated with poor patient outcome independent of *MYC* [49]. These data indicate that amplification of *CPSF1* may serve as an independent prognostic factor in cancer.

APA is widely dysregulated in cancer and can drive dysregulated gene expression, thus promoting oncogenic activity [3,6,7]. We showed that *CPSF1* amplification is associated with APA alterations in many cancer types. It is known that APA can impact gene expression and eventually affect cancer phenotype. We found an association between APA and dysregulated gene expression that are associated with cancer-related pathways. Some pathways are tumor-suppressive in nature and underwent APA lengthening coupled with decreased gene expression, supporting the idea that *CPSF1* amplification represses these pathways through APA. Conversely, oncogenic pathways underwent APA shortening coupled with increased gene expression. This is consistent with several studies showing global 3′UTR shortening of oncogenes. Of note, some pathways may be affected by *CPSF1* amplification on the gene expression level independent of APA, or vice versa. These findings highlight CPA copy number status as a potential regulator of APA and gene expression and warrant further investigation.

There are several limitations to this study that warrant further investigation. First, the low frequency of CPA mutations makes it difficult to discern the pathogenic impact of specific CPA variants. Therefore, specific CPA variants, especially those located at a domain with potential binding capacity to RNA, need further experimental investigation. Second, despite the clinical significance of *CPSF1* amplification in cancer, the biological role of this alteration is not known. Cell line models that harbor *CPSF1* amplification should be used to assess its impact on cancer cell phenotypes. Third, while we found an association between APA events and dysregulated gene expression, the functional effect of *CPSF1* amplification on the cleavage and polyadenylation process was not investigated. Future studies should investigate the dysregulation of alternative polyadenylation (APA) using functional genomics approaches. Such studies can lead to the discovery of specific APA-altered genes downstream of *CPSF1* that could have clinical relevance. Fourth, pan-cancer analyses can mask heterogeneity across individual tumor types and therefore future work should highlight cancer-context specific effects in individual cancer types. Finally, whether *CPSF1* amplification represents a therapeutic vulnerability is still an open question and will be investigated in future studies.

## 5. Conclusions

In conclusion, low prevalence of CPA gene mutations limits their prognostic value. In contrast, copy number alterations—most notably CPSF1 amplification—are associated with worse outcomes and APA shifting in a MYC-independent manner. These results nominate CPSF1 as a prognostic marker and a potential therapeutic target. Future work should investigate increased CPSF1 dosage effects experiments and assess whether CPSF1 targeting (both genetically and pharmacologically) reveals therapeutic vulnerabilities in CPSF1-amplified cancers.

## Figures and Tables

**Table 1:** Percentages of CPA gene copy number alterations in cancer.

Gene	Amplification	Gain	Diploid	Shallow Deletion	Deep Deletion
*CPSF1*	5.6	37.3	51	5.9	0.2
*CSTF1*	1.8	36.8	58	3.4	0
*CPSF4*	1.8	34.7	57.2	6.3	0
*CPSF6*	2.7	18.5	69.6	9.2	0
*SYMPK*	0.7	17.2	64.2	17.7	0.2
*CPSF3*	0.7	17.8	72.2	9.1	0.1
*NUDT21*	0.2	12.7	60.8	25.9	0.4
*CPSF2*	0.4	12.3	62.8	24.1	0.4
*CPSF7*	0.5	12.7	71.7	15	0.1
*CSTF3*	0.9	10.7	69.7	18.6	0.1
*CSTF2T*	0.3	9.3	65.5	24.5	0.4
*FIP1L1*	1.3	9.5	69	20.2	0.1
*CLP1*	0.3	12.3	72.2	15	0.1
*WDR33*	0.3	13.6	75.7	10.2	0.2

## Data Availability

The original data presented in the study are openly available through the following databases: the Genomic Data Commons (GDC) (https://gdc.cancer.gov/about-data/publications/pancanatlas) (accessed on 12 December 2024), cBioPortal for cancer genomics (www.cbioportal.org) (accessed on 25 January 2025), the UCSC Xena Browser (https://xenabrowser.net) (accessed on 25 January 2025), and The Cancer 3′UTR Atlas (TC3A) repository (https://github.com/CHENCANcc/TC3A_PDUI) (accessed on 5 August 2025). Codes used in this study, in general, were not original and were used from the publicly available R packages described in the Section 2. Specific modified codes are available by contacting the corresponding author.

## Supplementary Materials

The following supporting information can be downloaded at https://www.mdpi.com/article/10.3390/biology14121637/s1, Figure S1: The distribution of mutations across the *WDR33* CPA gene domains. Somatic mutation rate relative to all TCGA samples for *WDR33*. Y-axes indicate number of mutations for each specific variant; Figure S2: The association between mutation status and patient outcomes in the WDR33 CPA gene. Kaplan-Meier curves showing overall (A) and progression-free (B) survival for WDR33. “Blue” indicates patients with no mutation (wild type; “wt”) while “Red” indicates patients with mutations; “mut”; Figure S3: Pan-cancer landscape of copy number alterations in CPA genes. The oncoplot shows the percentages of copy number variations (CNV) in 16 CPA genes across 33 cancer types from TCGA. (A), All copy number alterations including amplification (red), gain (light red), shallow deletion (light blue), and deep deletion (blue). (B), Only gain (light red) and shallow deletion (light blue) CNVs are shown. Diploid (gray) denotes normal copy number; Figure S4: Representative APA altered genes. (A), Representative 3′UTR lengthening genes. (B), Representative 3′UTR shortening genes; Table S1: Number of mutations across various variant classes. Mutation counts are shown per CPA subcomplex.

## Abbreviations

The following abbreviations are used in this manuscript:

3′UTR	3′Untranslated Region
APA	Alternative polyadenylation
CCLE	Cancer Cell Line Encyclopedia
CFIIm	Mammalian cleavage factor II
CFIm	Mammalian cleavage factor I
CNV	Copy number variation
CPA	Cleavage and polyadenylation
CPSF	Cleavage and polyadenylation specificity factor
CSTF	Cleavage stimulation factor
MAF	Mutation Annotation Format
OS	Overall survival
PAS	Polyadenylation signal
PDUI	Percent Distal Usage Index
PFS	Progression-free survival
SNP	Single nucleotide polymorphism
TC3A	The Cancer 3′UTR Atlas
TCGA	The Cancer Genome Atlas
