# Supplementary material for: CPSF1 Is Co-Amplified with MYC but Is Independently Associated with Alternative Polyadenylation in Cancer
Source: Biology (Basel). 2025 Nov 21;14(12):1637. doi: 10.3390/biology14121637 (PMC12729778; doi:10.3390/biology14121637)
Supplement: Supplementary file 1 [file biology-14-01637-s001.zip › biology-3962447-Supplementary Figures.pptx]

## Slide 1
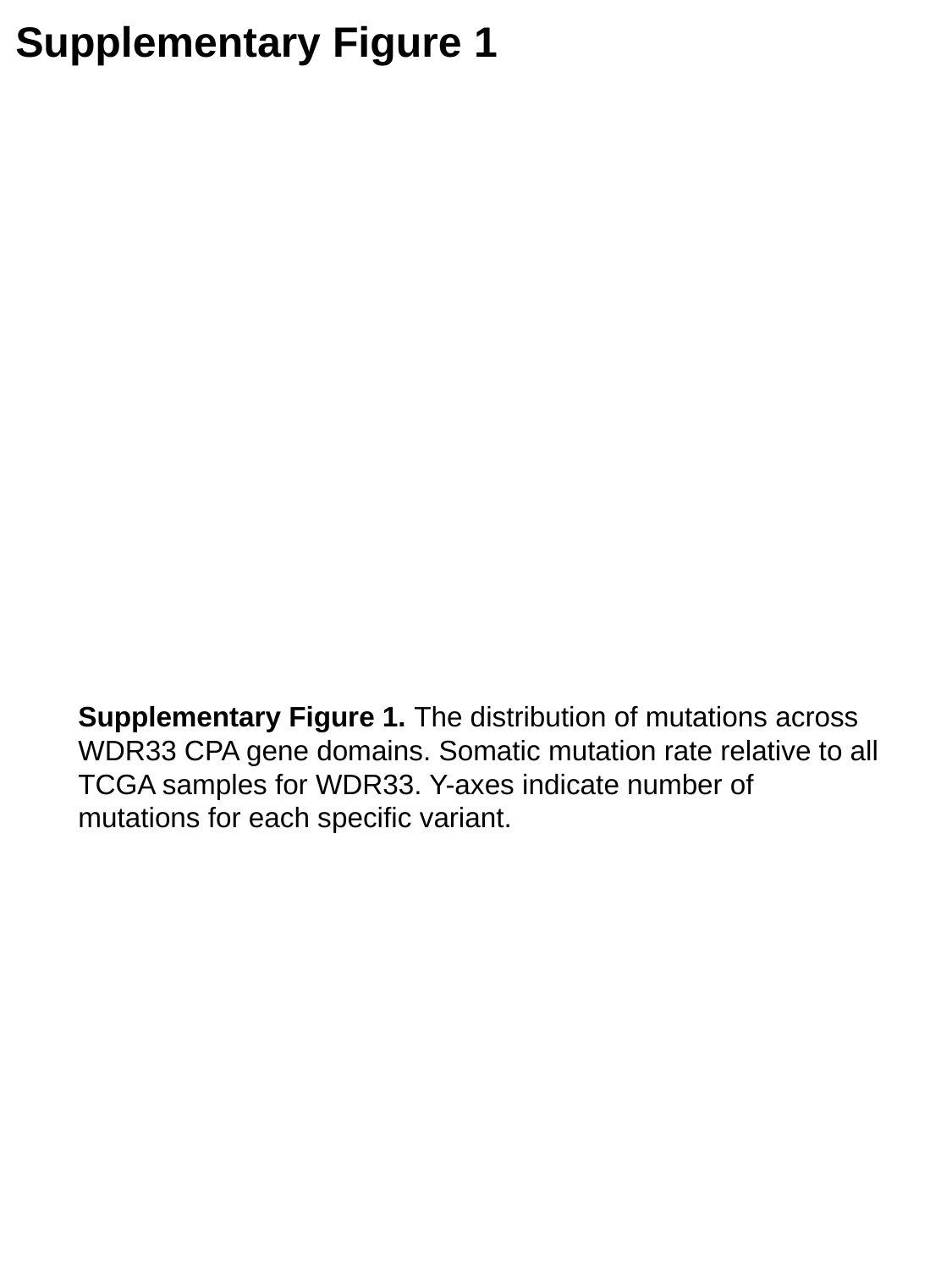

Supplementary Figure 1
Supplementary Figure 1. The distribution of mutations across WDR33 CPA gene domains. Somatic mutation rate relative to all TCGA samples for WDR33. Y-axes indicate number of mutations for each specific variant.

## Slide 2
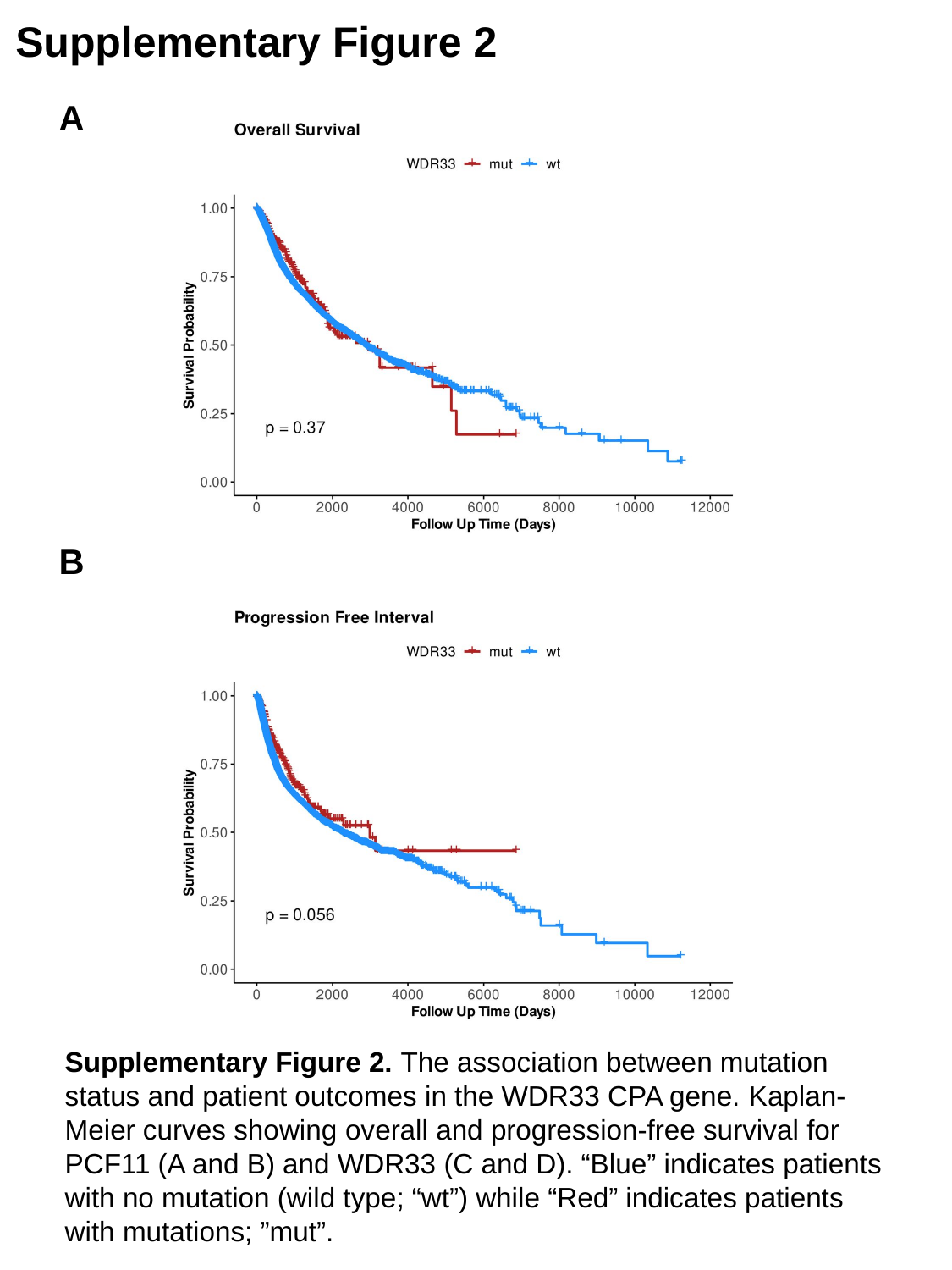

Supplementary Figure 2
A
B
Supplementary Figure 2. The association between mutation status and patient outcomes in the WDR33 CPA gene. Kaplan-Meier curves showing overall and progression-free survival for PCF11 (A and B) and WDR33 (C and D). “Blue” indicates patients with no mutation (wild type; “wt”) while “Red” indicates patients with mutations; ”mut”.

## Slide 3
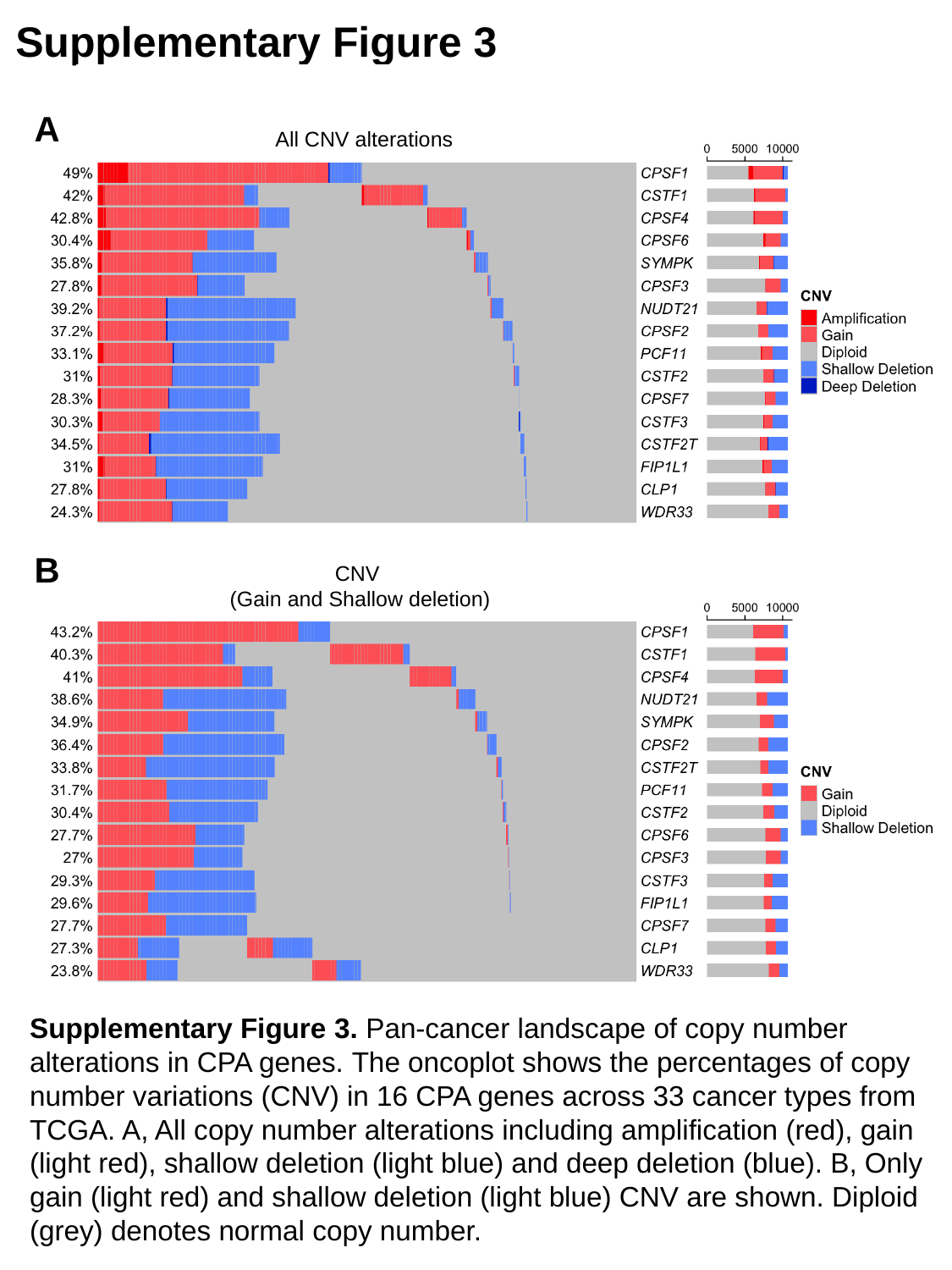

Supplementary Figure 3
All CNV alterations
A
CNV
(Gain and Shallow deletion)
B
Supplementary Figure 3. Pan-cancer landscape of copy number alterations in CPA genes. The oncoplot shows the percentages of copy number variations (CNV) in 16 CPA genes across 33 cancer types from TCGA. A, All copy number alterations including amplification (red), gain (light red), shallow deletion (light blue) and deep deletion (blue). B, Only gain (light red) and shallow deletion (light blue) CNV are shown. Diploid (grey) denotes normal copy number.

## Slide 4
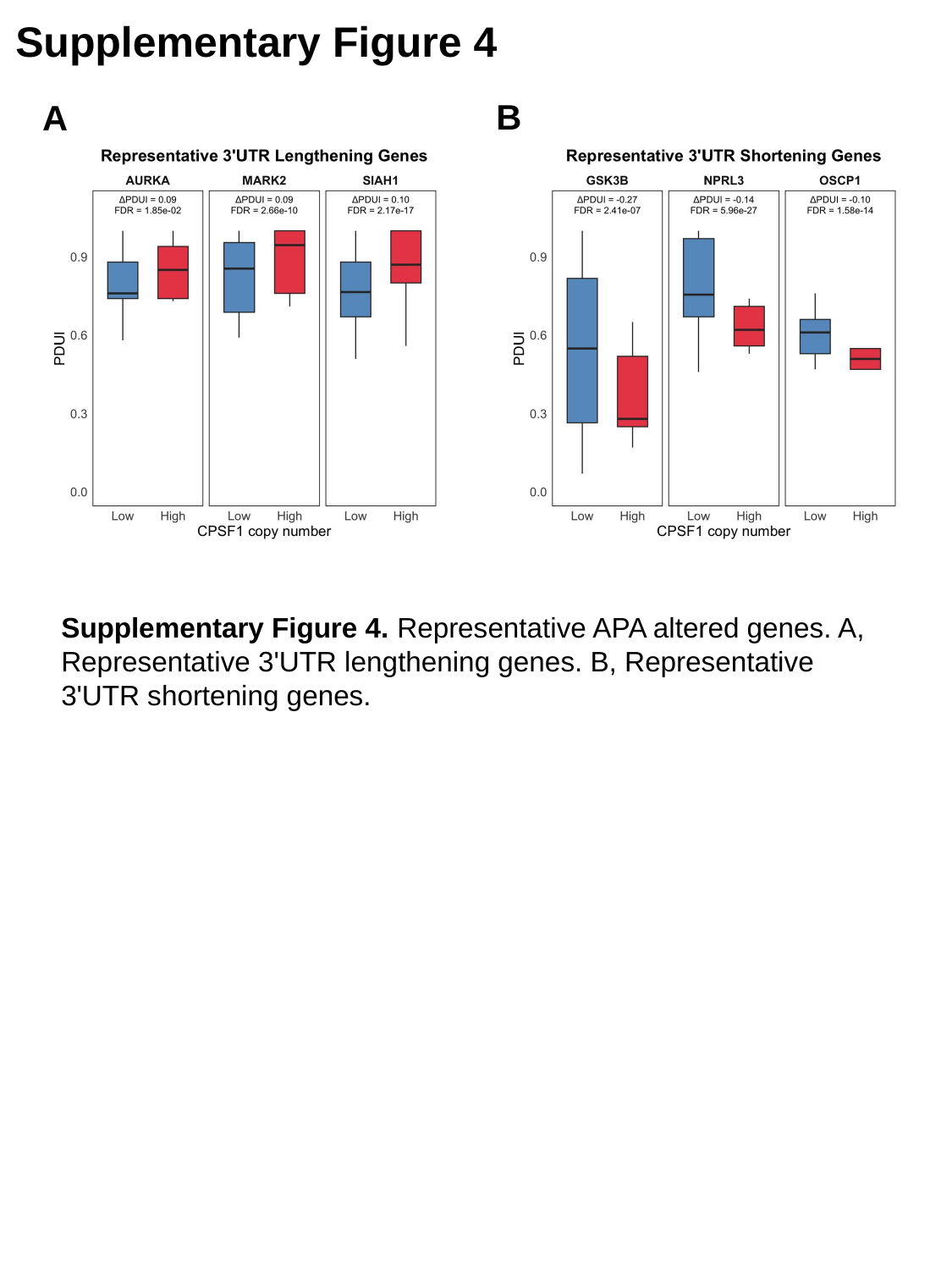

Supplementary Figure 4
B
A
Supplementary Figure 4. Representative APA altered genes. A, Representative 3'UTR lengthening genes. B, Representative 3'UTR shortening genes.
