# Supplementary material for: CPSF1 Is Co-Amplified with MYC but Is Independently Associated with Alternative Polyadenylation in Cancer
Source: Biology (Basel). 2025 Nov 21;14(12):1637. doi: 10.3390/biology14121637 (PMC12729778; doi:10.3390/biology14121637)
Supplement: Supplementary file 1 [file biology-14-01637-s001.zip › biology-3962447-Supplementary Table 1.pdf]

Supplementary Table S1. Number of mutations across various variant classes. Mutation counts are shown per CPA subcomplex.

Variant_Classification	CPSF	CFI	CFII	CSTF	Others	total	%
**Frame_Shift_Del**	37	9	17	24	37	124	5.247567
**Frame_Shift_Ins**	6	1	6	5	12	30	1.269573
**In_Frame_Del**	10	6	2	4	7	29	1.227253
**Missense_Mutation**	631	1	1	365	458	1456	61.61659
**Nonsense_Mutation**	63	195	303	21	42	624	26.40711
**Nonstop_Mutation**	1	11	44	1	0	57	2.412188
**Splice_Site**	14	1	2	10	16	43	1.819721
